# Generating and measuring the anisotropic elastic behaviour of Co thin films with oriented surface nano-strings on micro-cantilevers

**DOI:** 10.1186/1556-276X-6-325

**Published:** 2011-04-12

**Authors:** Vicente Madurga, José Vergara, Cristina Favieres

**Affiliations:** 1 Laboratory of Magnetism, Department of Physics, Public University of Navarre, Campus Arrosadía s/n, Pamplona 31006, Spain

## Abstract

In this research, the elastic behaviour of two Co thin films simultaneously deposited in an off-normal angle method was studied. Towards this end, two Si micro-cantilevers were simultaneously coated using pulsed laser deposition at an oblique angle, creating a Co nano-string surface morphology with a predetermined orientation. The selected position of each micro-cantilever during the coating process created longitudinal or transverse nano-strings. The anisotropic elastic behaviour of these Co films was determined by measuring the changes that took place in the resonant frequency of each micro-cantilever after this process of creating differently oriented plasma coatings had been completed. This differential procedure allowed us to determine the difference between the Young's modulus of the different films based on the different direction of the nano-strings. This difference was determined to be, at least, the 20% of the Young's modulus of the bulk Co.

PACS: 62.25.-g; 81.16.Rf; 68.60.Bs; 81.15.Fg; 68.37.Ef; 85.85.+j

## Introduction

The study of the elastic and mechanical properties of thin films is of interest in basic and applied research because thin films are used extensively in micro-electronic and micro-electromechanical systems. Because the elastic constants of thin films are different from those of bulk material of the same composition, the elastic constants of the bulk material cannot be used to design thin film devices. Consequently, it is very important to accurately determine the elastic constants of thin films. These properties can be studied using a wide variety of techniques, including the analysis of the substrate curvature [1], micro-beam testing [2], micro-tensile testing [3], cantilever-bending resonance [4], nano-indentation [5], Rayleigh-wave velocity measurements [6] and Brillouin scattering [7]. Among others, Young's modulus is an important parameter for thin-film technological applications.

Micro-cantilevers (MCLs) are mechanical devices with attractive applications; for instance, they are widely used as high-sensitivity sensors in different physical, chemical and biological technologies [8,9]. Another use of MCLs is in the study of the mechanical properties of thin films [10]. This type of analysis is possible because of the relation between the resonant frequency of MCLs and Young's modulus. If a MCL is coated with a thin film, a change results in the resonant frequency. By measuring this change, one can compute the Young's modulus of the thin film deposited on the MCL.

We conducted a study that demonstrated that the off-normal pulsed laser deposition (PLD) technique allows the simultaneous growth and sculpting of soft magnetic nano-strings with an orientation that is perpendicular to the incidence plane of the plasma and a medium width that can be selected between 8 and 30 nm by selecting an off-normal angle and the appropriate deposition time [11]. Uniaxial in-plane magnetic anisotropy was then generated in the films that would have a value between 10^3 ^and 10^4 ^J/m^3^, depending on the deposition parameters [11]. In addition to magnetic anisotropy, these nano-scale patterned Co films also presented controlled electrical, optical [12] and mechanical anisotropies [13]. In an extension of the study, MCLs were coated with these magnetic nano-strings so that their magneto-mechanical properties were analysed [14].

In this study, we produced Co nano-strings over Si MCLs, validating a differential method of studying the elastic anisotropy of these Co thin films in connection with their nano-string morphology. This technique allowed us to determine the difference between the Young's modulus of the films depending on their nano-string direction.

## Experimental procedures

**Figure 1:**
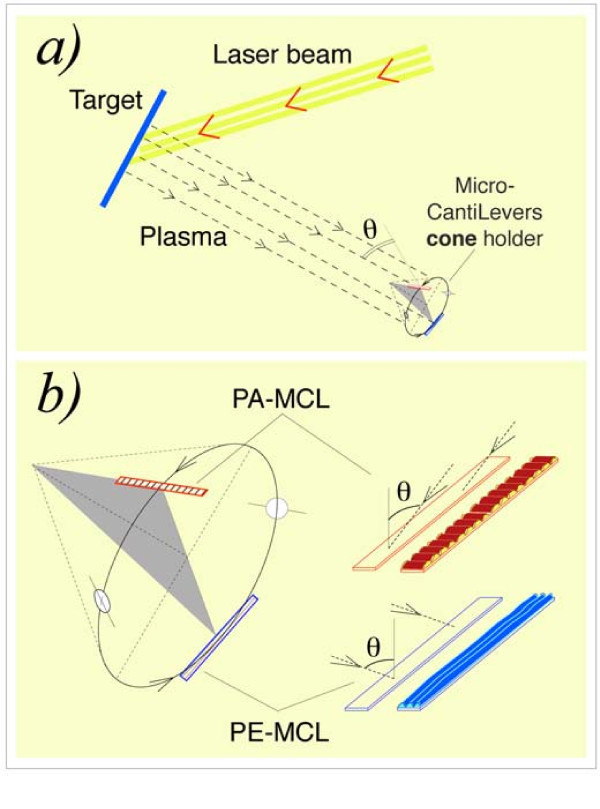
**Schematic representation of the device used in the simultaneous off-normal coating of the two micro-cantilevers**. **(a) **General view showing the plasma and the cone with the two MCLs. **(b) **(Left) Magnification of the cone with the MCLs: the PA-MCL is parallel to the cone generatrix, and the PE-MCL is perpendicular to the cone generatrix. Each MCL is located at one end of the diameter of a circle, which is a circular section perpendicular to the axis of the cone. Note that the two MCLs travel through the plasma in exactly the same circumference. **(b) **(Right) Schematic picture of the MCLs indicating the coating plasma direction and the transverse (PA-MCL) or longitudinal (PE-MCL) nano-strings generated.

Si MCLs, 450 × 50 × ≈ 2 μm^3 ^were coated with Co using PLD via an off-normal-incidence plasma procedure. A Nd:YAG laser beam (λ = 1054 nm, 20-Hz repetition rate, 240 mJ per 4.5-ns pulse, ≈12 GW, target spot area ≈12 mm^2^) was driven onto a pure, polished Co target located inside a chamber with a base pressure of 10^-6 ^mbar. The target rotated at 32 rpm and angle of the laser beam from normal to the target was 45°. The MCLs were positioned at a distance of 73 mm from the target and were placed on the lateral surface of a cone with an angle of π-2θ; the axis of the cone was parallel to the direction of the plasma to allow deposition at an off-normal angle, θ, as shown in Figure 1. In this study, the plasma generated reached two MCLs at an off-normal angle of θ = 55°. The cone rotated around its axis at 73 rpm. MCL holders were designed to allow the simultaneous off-normal coating of two MCLs, one parallel (PA-MCL) and one perpendicular (PE-MCL) to the generatrix of the cone, as shown in Figure 1b. Each MCL was located at each end of the diameter of a circle, a circular section perpendicular to the cone axis. Due to the cone rotation and the position of the two MCLs, the MCLs travelled through the plasma in exactly the same circumference, which ensured that each was coated with the same amount of material.

This designed, homemade device allowed the incidence plane of the plasma to be parallel or perpendicular to the longitudinal direction of each MCL. Therefore, the nano-strings generated in the off-normal deposited film were perpendicular (transverse) or parallel (longitudinal) to the longitudinal direction of each MCL, as shown in the right part of Figure 1b. In addition, two glass circles that were 7 mm in diameter were situated on the cone's lateral surface in the same circumference of the two MCLs. This made it possible to perform magnetic measurements.

The two MCLs were selected after the resonant frequency of each, ν_o_, had been determined. The two MCLs were similar because of their equal dimensions and because we did not allow differences between the frequencies of the two selected MCLs higher than 20 Hz in ≈10000 Hz. The two MCLs were simultaneously coated with Co in consecutive processes, either with the same coating time or with different coating times, whereas the rest of the parameters remained unchanged.

The same device was used to coat two MCLs with Au under the same conditions, which ensured that our device coated the two MCLs with the same amount of material.

The mechanical resonant frequency of the MCLs, ν_o _prior to coating and ν_(C-MCL) _after coating, was determined through location as the working MCL in the head of an atomic force microscope (AFM) [15]. The system performed a driving frequency scan for mechanical oscillation of the MCL, measuring the amplitude and the phase of the MCL's deflection. In this way, the MCL's resonant frequency, ν, was determined. The accuracy of the ν measurements was ± 1/10000.

Scanning tunnelling microscopy (STM) was performed to image the surface morphology of the coated glass circles and also the coated MCLs.

The magnetic hysteresis loops of the coated glass circles were determined using a vibrating sample magnetometer [14]. The value of the measured magnetic moment of each film was used to deduce its thickness. A deposition rate of ≈1.02 nm/min was used in this study. The different films had thicknesses between 0.25 and 28 nm.

## Results and discussion

**Figure 2:**
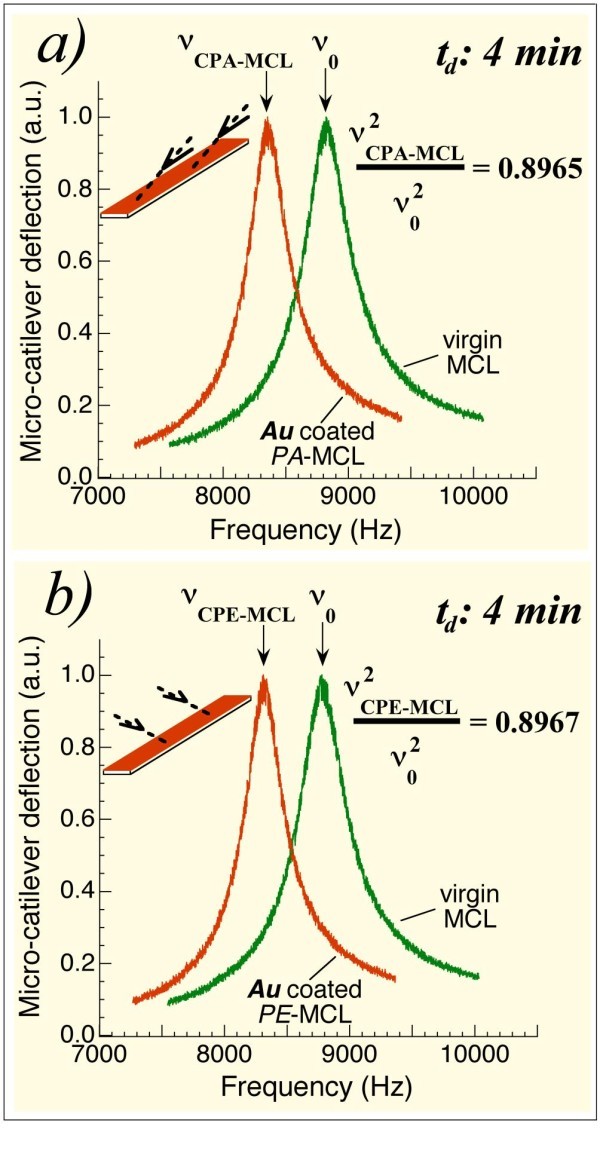
**Resonant frequencies of two simultaneously Au coated MCLs: isotropic elasticity of the films**. **(a) **Resonant frequency of an MCL before coating, ν_o_, and the corresponding frequency, ν_(CPA-MCL) _of the same MCL (now referred to as the CPA-MCL) after 4 min Au coating and positioned with its longitudinal direction parallel to the cone generatrix. **(b) **Resonant frequencies for the MCL prior to coating and the same MCL (now referred to as the CPE-MCL) after 4 min simultaneous Au coating and positioned with its longitudinal direction perpendicular to the cone generatrix. Note that the same value of the ratio ν^2^_(C-MCL)_/ν_o_^2 ^was measured for CPA-MCL and CPE-MCL. Evidence that the mass deposited on the PA-MCL is identical to that deposited on the PE-MCL is also shown.

Our previous studies of the surface morphology and physical properties of off-normal PLD Au thin films showed that no nano-strings, no electrical anisotropy and no optical anisotropy were generated in these samples. These results were different from those of off-normal PLD Co. Figure 2 shows the results for the two MCLs simultaneously coated with Au using deposition time *t*_d _= 4 min. The resonant frequencies of the MCLs before they were coated with Au, ν_o_, and afterwards, ν_(C-MCL)_, are indicated in this figure. The resonant frequency of a MCL before coating satisfies the expression ν_o_^2 ^~ *k*_o_/*m*_o _with *k*_o _the spring constant of the MCL and *m*_o _its mass. For the coated MCL, the C-MCL, the ratio ν^2^_(C-MCL)_/ν_o_^2 ^= (*k*_(C-MCL)_/*m*_(C-MCL)_)/(*k*_o_/*m*_o_) will vary when *k *or *m *changes: an increase in mass will decrease this ratio, and an increase in the spring constant will increase this ratio. For the PA-MCL, Figure 2a shows the difference between its resonant frequency, ν_o_, and its frequency after coating with its longitudinal direction parallel to the cone generatrix, frequency ν_(CPA-MCL)_. It is apparent that resonant frequency changes after coating, and the value of ν^2^_(CPA-MCL)_/ν_o_^2 ^is 0.8965. Figure 2b shows the corresponding results for the PE-MCL positioned with its longitudinal direction perpendicular to the cone generatrix. The corresponding frequency ratio is ν^2^_(CPE-MCL)_/ν_o_^2 ^= 0.8967. The measurements indicate that this ratio is equal for the two simultaneously Au-coated MCLs; the same shift in resonant frequency was detected. These first results suggest that no mechanical anisotropy was induced in the Au off-normal coated MCLs. Also, important evidence emerged indicating that the mass deposited on the PA-MCL was identical to that deposited on the PE-MCL. This last fact confirms that our system allows differential studies for both MCLs.

**Figure 3:**
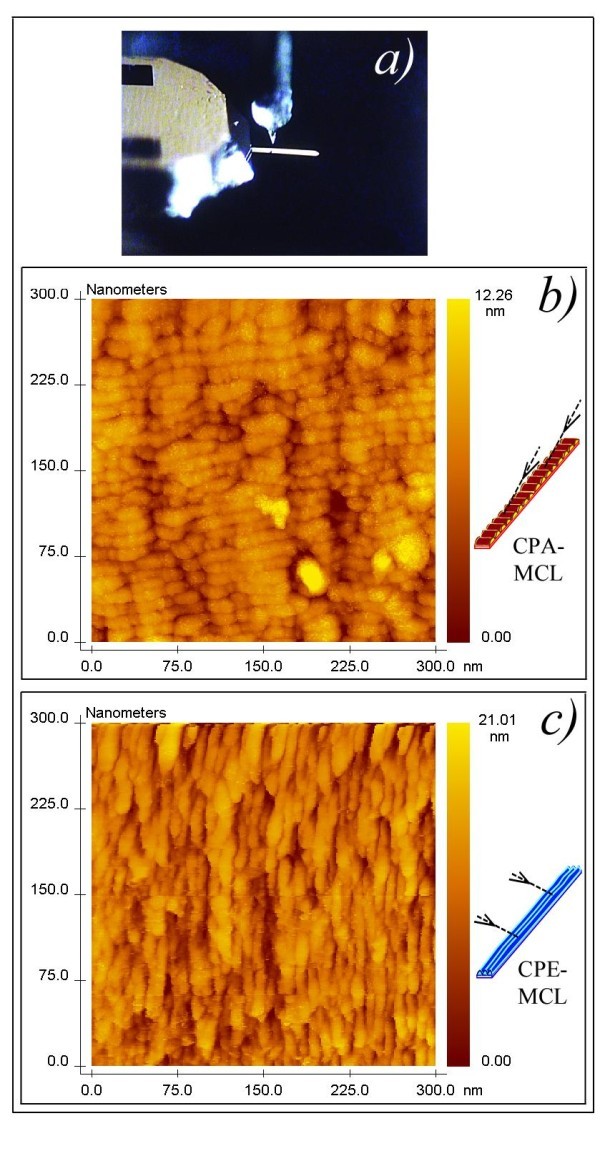
**Surface nano morphology of two simultaneously Co coated MCLs: different nano-strings were generated**. **(a) **Photo of a Co-coated MCL mounted in the STM sample holder for surface imaging. **(b) **STM image of the surface morphology of a Co-coated PA-MCL demonstrating the generation of the transverse nano-strings for the off-normal PLD. (**c) **STM image corresponding to the surface morphology of a Co-coated PE-MCL with the nano-strings in the longitudinal direction. These nano-scale patterns were visible in addition to the special elastic and mechanical properties of the MCLs.

The results for the off-normal Co-coated MCLs are different to those for the Au -coated MCLs. Figure 3b shows the surface morphology of a Co-coated PA-MCL, demonstrating the generation of the transverse nano-strings. Figure 3c shows the surface morphology of a Co-coated PE-MCL with longitudinal nano-strings. The average width of the nano-strings was 12 nm. This nano-scale patterned was correlated with the elastic and mechanical properties of the MCLs, as shown in the next results.

**Figure 4:**
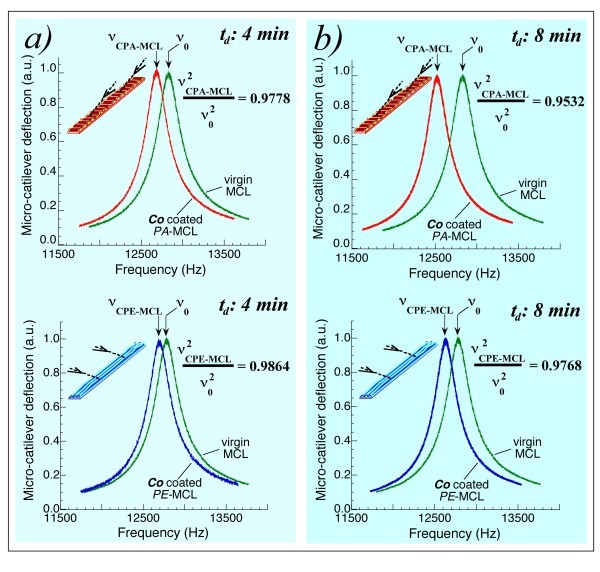
**Resonant frequencies of two simultaneously Co coated MCLs: anisotropic elasticity of the films**. **(a) **(Top) Resonant frequencies for the PA-MCL: ν_o _representing the resonant frequency before the coating process and ν_(CPA-MCL) _representing the resonant frequency after the Co coating process with deposition time *t *= 4 min. ν^2^_(CPA-MCL)_/ν_o_^2 ^= 0.9778. (Bottom) Resonant frequencies for the simultaneously coated PE-MCL: ν_o _representing the resonant frequency prior to the coating process and ν_(CPE-MCL) _representing the resonant frequency after the coating process; ν^2^_(CPE-MCL)_/ν_o_^2 ^= 0.9864. **(b) **Resonant frequencies of the two simultaneously coated MCLs coated consecutively for 4 min: that is, for a total deposition time of 8 min. ν^2^_(CPA-MCL)_/ν_o_^2 ^= 0.9532 and ν^2^_(CPE-MCL)_/ν_o_^2 ^= 0.9768. Note the significant difference between the PA-MCL and PE-MCL ratios for the two cases and the difference between these results and those displayed in Figure 2.

The top of Figure 4a shows the resonant frequencies of the PA-MCL: ν_o_, before the coating process and ν_(CPA-MCL) _after the coating process for a deposition time *t *= 4 min. For this coated PA-MCL, the ratio ν^2^_(CPA-MCL)_/ν_o_^2 ^is 0.9778. The PE-MCL, simultaneously coated with the PA-MCL, also exhibited a shift in its resonant frequency such that ν^2^_(CPE-MCL)_/ν_o_^2 ^= 0.9864, as shown on the bottom of Figure 4a. Unlike the two Au-coated MCLs (for which the two ratios were equal: 0.8965 and 0.8967), these two simultaneously Co-coated MCLs exhibited different mechanical behaviour depending on the position of the cantilever during the coating process; when the MCL was parallel to the cone generatrix, the PA-MCL, the ratio was 0.9778, and when the MCL was perpendicular to the cone generatrix, the PE-MCL, the ratio was 0.9864. This effect remained when the deposition time increased. Figure 4b shows the results when the two simultaneously coated MCLs were consecutively coated for other 4 min; that is, for a total deposition time of 8 min. Having demonstrated that the amount of material deposited onto each MCL was equal, we can remark that the spring constant of each Co-coated PA- or PE-MCL changed according to the longitudinal or transverse orientation of the film's nano-strings.

**Figure 5:**
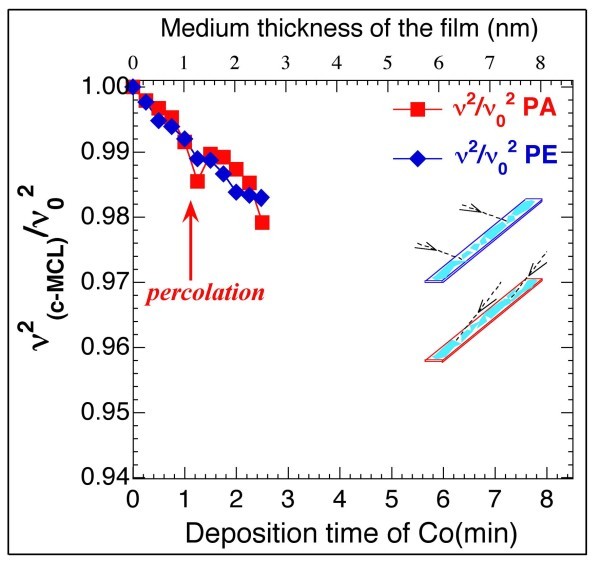
**Evolution of the ratio ν^2^_(C-MCL)_/ν_o_^2 ^with a consecutive Co deposition time of 15 s**. This ratio is shown for both the PA-MCL and the PE-MCL. The percolation in the deposited Co over the MCLs was deduced for a total deposition time of ≈1.2 to 1.4 min when ν^2^_(C-MCL)_/ν_o_^2 ^changed its slope.

Figure 5 shows the changes in the ratio ν^2^_(C-MCL)_/ν_o_^2 ^with consecutive Co deposition times of 15 s. Ratios are displayed for both the PA-MCL and the PE-MCL. These results indicate that there is no difference between the PA- and PE-MCL with regard to these parameters until ≈1.0 min and that the same decrease occurs for both with time. The lack of difference may stem from the equal mass deposited on both MCLs and the equal *k*_0 _spring constants for both MCLs. No film was formed, only islands of Co were present and no change of the *k*_0 _of each MCL took place. After percolation, after ≈1.2-1.4 min of deposition, the slope of the ratio ν^2^_(C-MCL)_/ν_o_^2 ^versus the deposition time, changed. The decrease in ν^2^_(C-MCL)_/ν_o_^2 ^produced by the increase in *m *was balanced out by the increase in *k *produced by the percolated film. Because the same quantity of material was deposited on the two simultaneously coated MCLs, the division of the value of ν^2^_(C-MCL)_/ν_o_^2 ^(starting at approximately 2.0 min) must has been a result of the newly generated nano-strings, which produced different values of *k *for each MCL. In fact, the coated PE-MCL with longitudinal nano-strings exhibited a value of *k *higher than the corresponding value for the coated PA-MCL with transverse nano-strings.

**Figure 6:**
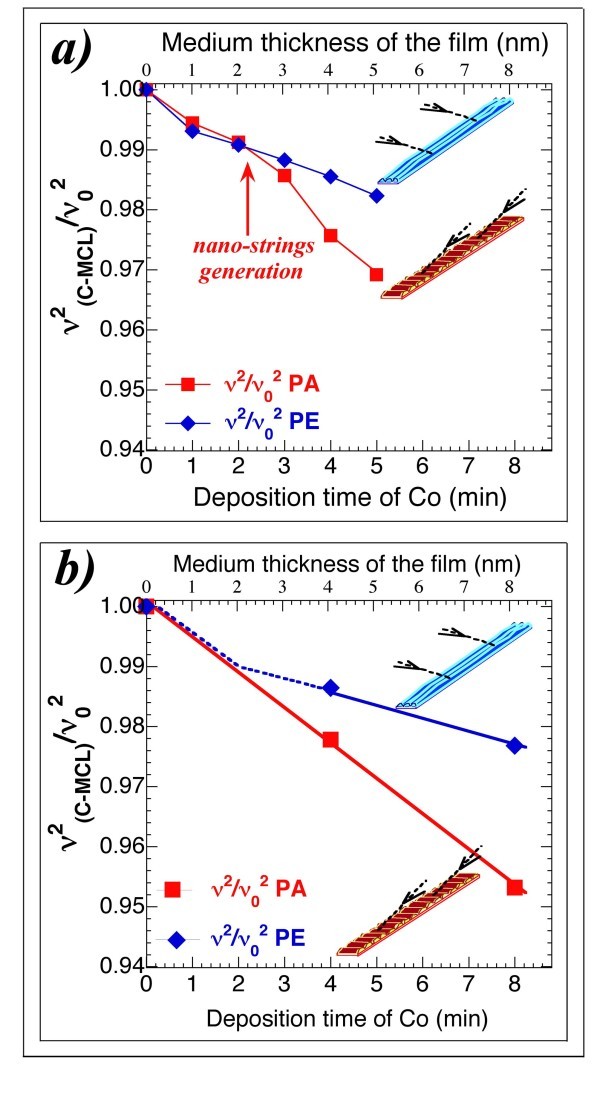
**Evolution of the ratio ν^2^_(C-MCL)_/ν_o_^2 ^with consecutive Co deposition times**. **(a) **Evolution of the ratio ν^2^_(C-MCL)_/ν_o_^2 ^with a consecutive deposition time of 1.0 min. This ratio for the CPA-MCL (featuring transverse nano-strings) has a slope that is practically equal to its initial slope and is consistent with the increment in mass of the C-MCL. This slope for the coated CPE-MCL (longitudinal nano-strings) is smaller than the slope for the CPA-MCL: an increase in the value of its spring constant, *k*_o_, must have occurred because the increase in mass was the same for the two MCLs. **(b) **The same behaviour was observed for the other two simultaneously off-normal Co-coated micro-cantilevers with consecutive deposition times of 4.0 min.

At higher deposition times, when the nano-strings had begun to grow successfully, the difference between the mechanical behaviour of the simultaneously off-normal coated PA- and PE-MCLs increased, as shown in Figure 6. The changes in the ratio ν^2^_(C-MCL)_/ν_o_^2 ^with a Co consecutive deposition time of 1.0 min (see Figure 6a) show how this ratio for the CPA-MCL (featuring the transverse nano-strings) has a slope practically equal to its initial slope and consistent with the increase in mass of the MCL. The slope for the CPE-MCL (with longitudinal nano-strings) is lower than the slope for the CPA-MCL, and because the increase in mass was equal for both MCLs, an increase in the value of the spring constant, *k*_0_, must have occurred for the CPE-MCL. One preliminary conclusion can be made: the off-normal Co-coating process increased the spring constant of the MCL with longitudinal nano-strings, whereas for the MCL with transverse nano-strings, which was coated during the same process, only small changes of its spring constant occurred.

This behaviour was also observed for other two simultaneously off-normal Co-coated MCLs with a consecutive deposition time of 4.0 min, as shown in Figure 6b.

Taking into account [16,17] that(1)

being *C *= 1.875 and the resonant frequencies ν_o _(in the interval (8665 ± 5) Hz), the density of the Si (ρ_0 _= 2.33 × 10^3 ^kg/m^3^), the Si Young's modulus (*E*_0 _= 1.69 × 10^11 ^Pa), and the length (*L *= 450 μm) and width (*w *= 50 μm) of the two MCLs in Figure 6a, the following values were deduced for MCL: mass, *m*_o _= 6.66 × 10^-11 ^kg, *k*_o _= 0.200 N/m and thickness *t *= 1.7 μm. The resonant frequency of a coated MCL is [16,17]:(2)

where δ is the thickness of the deposited Co film and *E *its Young's modulus. Considering that δ = 10 nm, that *t *≈ 2000 nm, that *w *= 50000 nm and that the two MCLs are practically equal, we have approximated this last equation, resulting:(3)

with *E*_lng _and *E*_trs _the Young's modulus of the CPE film and CPA film, respectively, and 1.025 the experimental value of the MCLs in Figure 4b. Working from this last equation, we obtain the following:(4)

Given the *E*_0 _value, this difference is ≈20% of the Young's modulus of the micro-crystalline hcp bulk Co.

## Conclusions

A specially designed homemade device combined with a PLD system allowed the off-normal simultaneous coating of two Si MCLs at different controlled locations with respect to the incidence plane of the plasma. For a fixed off-normal angle of θ = 55°, two positions were used for the two MCLs: a position parallel to the incidence plane of the plasma and one perpendicular to that plane. The two off-normal Au-coated MCLs exhibited equal mechanical behaviour, indicating the in-plane isotropic elasticity of these Au pulsed-laser deposited films. This equal mechanical behaviour ensured that the amount of material deposited on both simultaneously coated MCLs was equal and made it possible to conduct a differential analysis between both. The two simultaneously off-normal Co-coated MCLs exhibited the following behaviour. First, after percolation and nano-string generation, different mechanical behaviour occurred due to the increase in the spring constant for the MCL with Co nano-strings parallel to the longitudinal direction, whereas the MCL with Co nano-strings transverse to the longitudinal direction experienced changes in the resonant frequency mostly produced by the increase in mass. Secondly, these results were connected with the anisotropic elastic behaviour of the Co film with nano-strings morphology. Thirdly, the Young's modulus of the off-normal deposited Co film was 20% of the Young's modulus of the bulk Co higher for the film direction parallel to the nano-strings than for the film direction transverse to the nano-strings.

## Abbreviations

AFM: atomic force microscope; C-MCL: coated micro-cantilever; CPA: film deposited over the microcantilever parallel to the cone generatrix; CPE: film deposited over the microcantilever perpendicular to the cone generatrix; CPA-MCL: coated micro-cantilever parallel to the cone generatrix; CPE-MCL: coated micro-cantilever perpendicular to the cone generatrix; C(PA or PE)-MCL: coated micro-cantilever parallel to the cone generatrix or coated micro-cantilever perpendicular: to the cone generatrix; MCLs: micro-cantilevers; Nd:YAG: neodymium-doped yttrium aluminium garnet; PA-MCL: micro-cantilever parallel to the cone generatrix; PE-MCL: micro-cantilever perpendicular to the cone generatrix; PLD: pulsed laser deposition; STM: scanning tunnelling microscopy.

## Competing interests

The authors declare that they have no competing interests.

## Authors' contributions

VM, CF and JV participated from the beginning in devising the different steps of the work. Specially, VM with the preparation of the device for off-normal PLD, supports and microcantilever holders and during the coating processes. CF with mechanical characterization of microcantilever and subsequent determination of mechanical resonances. JV with the STM surface observation nano-strings of the microcantilever and VSM magnetic determinations. VM, CF and JV participated at the discussions and analysis of the results and during the preparation of manuscript. Specially CF dedicated extra time for this part.

## References

[B1] Nix WD Mechanical properties of thin films Metall Trans A Phys Metall Mater Sci 1989 20A 2217 2245 10.1007/BF02666659

[B2] Florando JN Nix WD A microbeam bending method for studying stress-strain relations for metal thin films on silicon substrates J Mech Phys Solids 2005 53 619 638 10.1016/j.jmps.2004.08.007

[B3] Badawi KF Villain P Goudeau Ph Renault PO Measuring thin film and multilayer elastic constants by coupling in situ tensile testing with x-ray diffraction Appl Phys Lett 2002 80 4705 4707 10.1063/1.1488701

[B4] Yamaguchi T Song W Yamaguchi A Yamamoto R The anelastic study of Ag/Pd multilayers J Alloys Compd 1994 211-212 442 445 10.1016/0925-8388(94)90540-1

[B5] Chen X Vlassak J Numerical study on the measurement of thin film mechanical properties by means of nanoindentation J Mater Res 2001 16 2974 2982 10.1557/JMR.2001.0408

[B6] Danner R Huebener RP Chun CS Grimsditch M Schuller IK Surface acoustic waves in Ni/V superlattices Phys Rev B 1986 33 3696 3701 10.1103/PhysRevB.33.3696 9938778

[B7] Mirkarimi PB Shinn M Barnett SA Kumar S Grimsditch M Elastic properties of TiN/(V_x_Nb_1-x_)N superlattices measured lby Brillouin scattering J Appl Phys 1992 71 4955 4958 10.1063/1.350644

[B8] Craighead HG Waggoner PS Micro- and nanomechanical sensors for environmental, chemical, and biological detection Lab Chip 2007 7 1238 1255 10.1039/b707401h 17896006

[B9] Finot E Passian A Thundat T Measurement of mechanical properties of cantilever shaped materials Sensors 2008 8 3497 3541 10.3390/s8053497 PMC3675557 27879891

[B10] McShane GJ Boutchich M Srikantha Phani A Moore DF Lu TJ Young's modulus measurement of thin-film materials using micro-cantilevers J Micromech Microeng 2006 16 1926 1938 10.1088/0960-1317/16/10/003

[B11] Madurga V Vergara J Favieres C Magnetic domain structures and nano-string morphology of laser off-normal deposited amorphous cobalt films with controlled magnetic anisotropy J Magn Magn Matter 2004 272-276 1681 1683 10.1016/j.jmmm.2003.12.251

[B12] Madurga V Vergara J Favieres C Surface nano-string morphology of oblique pulsed laser deposited cobalt thin films International Conference TNT "Trends in Nanotechnology": 29 August-2 September 2005, Oviedo, Spain

[B13] Madurga V Vergara J Favieres C Soft magnetic nano-strings simultaneously grown and sculpted on Si micro-cantilevers J Magn Magn Matter 2010 322 1519 1522 10.1016/j.jmmm.2009.11.036

[B14] Madurga V Favieres C Vergara J Growth and sculpting of Co nano-strings on Si micro-cantilevers: magneto-mechanical properties Nanotechnology 2010 21 095702 6 10.1088/0957-4484/21/9/095702 20124658

[B15] Horcas I Hernández R Gómez-Rodríguez JM Colchero J Gómez-Herrero J Baró A WSXM: A software for scanning probe microscopy and a tool for nanotechnology Rev Sci Instrum 2007 78 013705 8 10.1063/1.2432410 17503926

[B16] Bishop RDE Johnson DC The Mechanics of Vibration 1960 Cambrigde:Cambridge University Press

[B17] Salvadori MC Grown IG Vaz AR Melo LL Cattani MC Measurement of the elastic modulus of nanostructured gold and platinum thin Films Phys Rev B 2003 67 153404 4 10.1103/PhysRevB.67.153404

